# Finnish Youth Footballers' Perceptions on Artificial Turf: A Survey Research

**DOI:** 10.1002/hsr2.71594

**Published:** 2025-11-27

**Authors:** Ville Immonen, Matias Vaajala, Oskari Pakarinen, Lauri Nyrhi, Ilari Kuitunen

**Affiliations:** ^1^ University of Eastern Finland, Institute of Clinical Medicine Kuopio Finland; ^2^ Tampere University Tampere Finland; ^3^ Päijät‐Häme Central Hospital Lahti Finland; ^4^ Central Finland Hospital Nova, Department of Surgery Jyväskylä Finland; ^5^ Department of Pediatrics Kuopio University Hospital Kuopio Finland

**Keywords:** artificial turf, Football, injury, playing surface, youth

## Abstract

**Background and Aims:**

There is no evidence of increased overall injury risk on modern artificial surfaces in football. However, football players have generally considered artificial surface to increase injury risk compared to natural grass. Earlier studies about football players' perceptions on playing surfaces have been conducted using adults who primarily play on natural grass. This study aimed to examine Finnish youth players' perceptions on artificial turf.

**Methods:**

An online questionnaire form was sent to three Finnish clubs (Mikkelin Palloilijat, FC Inter Turku and Vantaan Jalkapalloseura). The questionnaire consisted of players' basic information and ten multiple‐choice questions. Questions considered both injuries and players' preferences on artificial turf and natural grass. Descriptive statistics (frequencies, percentages, means and standard deviations) were calculated. Differences in proportions were tested using a two‐proportion *Z*‐test.

**Results:**

A total of 136 youth players (11–20 years old) answered the questionnaire. Most respondents played football primarily on artificial turf. Nearly half of the respondents agreed that injuries are more common on artificial turf compared to natural grass. Of 11–15‐year‐olds, 58% agreed with this statement while only 19% of 16–20‐year‐olds agreed (*z* = 3.19, *p* = 0.001). Over a half of the respondents assessed knee injuries and overuse injuries to happen more likely on artificial turf. More respondents favored natural grass as a primary match surface but there was no difference in preference considering training surface.

**Conclusion:**

Finnish youth football players consider injury risk to increase on artificial turf. Based on our study, this opinion is related to players' age. Many perceptions on artificial turf were relatively positive compared to previous studies, which could be explained with the familiarity of the surface among the respondents.

## 1 Background and Aims

Natural grass is the primary surface in football in most parts of the world. Compared to natural surfaces, artificial turf offers multiple practical advantages such as weather resistance and higher usage rates. The International Association Football Federation (FIFA) defines artificial turf as the best alternative to natural grass [1]. Due to significant advancements in artificial materials, the use of artificial surfaces has become increasingly common over the years [2].

There is no evidence of an increased overall injury risk on modern artificial pitches in football, and recent analyses even indicate that the overall injury risk in football is increased on natural grass [3, 4]. In contrast, some studies have shown artificial turf to increase injuries in specific subgroups [5, 6]. There have been multiple public claims against the safety of artificial surfaces from professional footballers and coaches in recent years [7, 8, 9, 10]. After the 2015 Women's World Cup in Canada – where artificial turf was notably used in an international tournament – players launched a discrimination case against FIFA claiming that the concern of injuries on artificial turf impacts the game negatively [11].

Previous studies about footballers' perceptions on artificial turf have been conducted with amateur [12, 13] and professional players [14, 15, 16, 17, 18, 19, 20, 21]. Professional players have generally considered artificial turf as a major injury factor [15, 16, 20, 21]. To our knowledge, no previous survey research on youth football players' perceptions on artificial turf have been performed. Previous studies are also mostly made in areas where artificial turf is not a primary playing surface, and only one previous study about football players' impressions has been made in Nordic countries [17]. This particular study was made with Swedish elite‐level footballers. No previous surveys for Finnish footballers have been performed.

This study aimed to examine Finnish youth (11‐20 years old) football players' perceptions on artificial turf. Injuries were the primary focus of the study. The main hypothesis of the study was that youth players would evaluate the injury risk to be higher on artificial turf.

## 2 Materials and Methods

### 2.1 Study Design

This was a survey research. There was no standardized questionnaire form available for the purpose of this study. An original online form was created using the Webropol‐application. The initial form was reviewed by a professional communications specialist and the edited form was further tested with 24 Finnish adult (20‐25 years old) amateur footballers. The final form was created based on open feedback from these respondents.

### 2.2 Study Population

A link to the questionnaire was sent via email to three participating youth football clubs: Mikkelin Palloilijat (MP), FC Inter Turku, and Vantaan Jalkapalloseura (VJS). Two of these clubs (Inter and VJS) are among ten largest football clubs in Finland by registered members, both with approximately 1700 licensed players (youth and adult players included). MP is a relatively smaller club with approximately 500 licensed players. Inter is currently the only one of these clubs with a first team in the premier division of Finland, both males and females included.

We did not specify the details of the pitches the clubs were using at the time of the study. Artificial surfaces in Finland used in football are generally at least third‐generation technology systems containing both sand and rubber infill. Every participating club has access to artificial turf (inside and outside) all year. According to club websites, VJS and Inter have a heating system on at least one of their artificial pitches making it possible to use them in winter time. VJS also has their own football arena with artificial turf, while Inter and MP are using public inside facilities in winter. In summer, VJS and MP report to have their own natural grass surface available while Inter does not separately mention this on their club website. Teams did not report the timespan they had access to natural grass. Typically, natural grass is available for football from May to October in southern parts of Finland during the Finnish premier division season. All of these clubs are located in the southern parts of Finland and were not considered to face significant climatic differences at their home pitches.

We included answers from players aged 11–20 years to focus on youth respondents. The age range was chosen based on a hypothesis that older than ten‐year‐olds can understand and answer the questions with sufficient precision (a similar age criterion is used in Finnish governmental school healthcare questionnaires) and 20 is the oldest age limit to play in youth divisions in Finland.

### 2.3 Data Collection

Responses were collected from February 14th to March 31st 2023. Age, gender, position, football experience, and current club were reported. Players also reported their current primary training surface in winter, spring, summer and autumn. Respondents were categorized into two age groups (11–15‐year‐olds and 16–20‐year‐olds), three gender groups (male, female and other), four positional groups (attacker, midfielder, defender, goalkeeper) and three experience groups (0–5 years, 6–10 years and more than ten years of playing football). In positional grouping, players were allowed to report their playing position as undefined.

The main questionnaire consisted of 11 questions which considered both injuries and players' preferences or observations towards playing surfaces. A response was included in the study if all the questions were answered and the respondent fulfilled the age criteria. One question from the final questionnaire form (favored stud type on artificial turf) was eventually not included in the study since it was considered to fall outside the aim of the study. Therefore, ten questions were eventually included in the results. All the questions were close‐ended, multiple‐choice questions with only one answer allowed per question.

In first five questions, respondents were asked how much they agreed with a statement using five‐level Likert scale (disagree, disagree on some level, neutral, agree on some level or agree). The five statements were:

“Injuries are more common on artificial turf compared to natural grass”.

“I am afraid of injuries on artificial turf”.

“I am afraid of injuries on natural grass”.

“Quality of game is worse on artificial turf compared to natural grass”.

“Artificial turf should not be used if natural grass is available”.

The responses were dichotomized (agree/agree on some level *vs.* disagree/disagree on some level/neutral) for more informative outcome. Separate results for age groups were reported.

In sixth question, respondents were asked to assess how much a certain factor has influence on overall injury risk in football using five‐level Likert scale (no effect, little effect, some effect, a lot of effect, truly a lot of effect). The factors in question were warm‐up, temperature, raining, snowing, playing surface, shoe material, stud type, player's weight and length, growth spurt, previous injuries and muscle strength.

In the last four questions respondents were asked about differences and preferences between artificial turf and natural grass. Respondents were able to choose one of these surfaces or a neutral “no difference/preference” option. The four questions were:

“Which surface causes more injuries in the category”. The categories in question were anatomical regions (ankle, knee, thigh, pelvis, upper extremity) or injury types (acute, overuse).

“Preferred surface for the football action”. The actions in question were match play, training, running, shooting, passing, sliding, first touch and ball control.

“Preferred match surface depending on the month”. In this question, artificial turf option was categorized to inside and outside turf.

“Preferred match surface from best to worst”. In this question, surfaces were categorized to good and bad based on the subjective pitch condition.

### 2.4 Statistical Analysis

Statistical analysis was performed using Microsoft Excel and R version 4.0.5. Responses were tabled and descriptive statistics (frequencies, percentages, means and standard deviations) were calculated. Differences in proportions were tested using a two‐proportion Z‐test. *p*‐values under 0.05 were considered statistically significant.

### 2.5 Ethical and Consent Statement

Permission to distribute the questionnaire to players was obtained from the governance of the participating clubs. Link to the questionnaire form was distributed by the clubs' staff. All respondents were informed about the purpose and the usage of the data before the study. All the respondents were asked to provide a consent for participation. Participation was voluntary and information was collected anonymously. According to Finnish regulations, since health information or other sensitive data was not gathered from the respondents, an approval by an ethics committee was not required. According to Finnish research laws, parents' permission is required from respondents under 15 years of age. We asked the players to inform their legal guardians about this survey and provide their guardians' email address as proof of consent.

## 3 Results

A total of 528 players opened the questionnaire, of whom 273 started filling it and 176 completed it. Of these 176 responses, 40 were either incomplete or the player did not match the age criteria. Therefore, a total of 136 responses were included in the study. Of the included respondents, 69 played for Inter, 47 for MP and 20 for VJS. The age groups were approximately evenly represented. The majority of respondents were male and had more than 5 years of football experience. All playing positions were represented among the respondents (Table 1).

**Table 1:** Background characteristics of the respondents (*n* = 136).

	*n*	%
*Team*		
Mikkelin Palloilijat (MP)	47	35
FC Inter Turku	69	51
Vantaan Jalkapalloseura (VJS)	20	15
*Age (mean 15, sd 2)*		
11–15 years	71	52
16–20 years	65	48
*Gender*		
Male	127	93
Female	9	7
Other	0	0
*Position*		
Attacker	27	20
Midfielder	36	26
Defender	40	29
Goalkeeper	18	13
Undefined	15	11
*Football playing years (mean 9, sd 3)*		
0–5 years	10	7
6–10 years	89	65
10+ years	37	27

**Figure 1:**
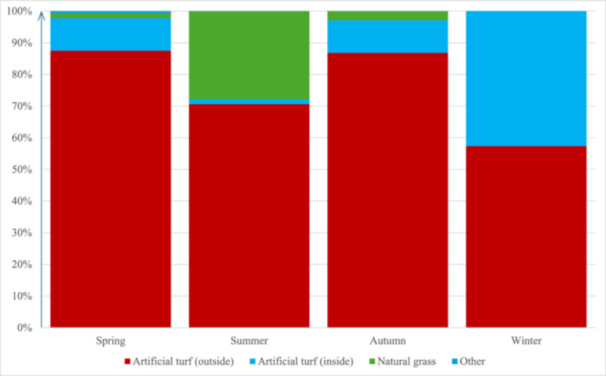
Primary training surface depending on the season (*n* = 136).

Outdoor artificial turf was the most commonly used training surface among the respondents regardless of the season. During summer, 38 respondents (28%) trained mainly on natural grass, while during winter, 58 respondents (43%) trained indoors on artificial turf (Figure 1, Supplementary Table 1).

Fifty‐nine respondents (43%) agreed at least on some level that injuries are more common on artificial turf than on natural grass. There was a significant difference between age groups: 58% of 11–15‐year‐olds agreed at least on some level with the statement, while only 19% of 16–20‐year‐olds agreed at least on some level (*Z* = 3.2, *p* = 0.001) (Table 2).

**Table 2:** Opinions on the statements related to injuries on artificial turf and natural grass overall (*n* = 136), for 11–15‐year‐olds (*n* = 71), and for 16–20‐year‐olds (*n* = 65). The distribution opinion between the age groups was compared using *Z*‐score with *p*‐values.

	All players		11–15 years			16–20 years			
	*n*	%		*n*	%	*n*	%	*Z*‐score	*p*‐value
*Injuries are more common on artificial turf than on natural grass*									
agree/agree on some level	59	43		40	58	19	29	3.2	0.001
disagree/disagree on some level/neutral	77	57		31	42	46	71		
*I am afraid of injuries on artificial turf*									
agree/agree on some level	12	9		4	56	8	12	−1.4	0.17
disagree/disagree on some level/neutral	124	91		67	44	57	88		
*I am afraid of injuries on natural grass*									
agree/agree on some level	18	13		6	8	12	18	−1.7	0.09
disagree/disagree on some level/neutral	118	87		65	92	53	82		
*Quality of game is worse on artificial turf*									
agree/agree on some level	28	21		10	14	18	28	−2.0	0.05
disagree/disagree on some level/neutral	108	79		61	86	47	72		
*Artificial turf should not be used if natural grass is available*									
agree/agree on some level	67	49		30	42	37	57	−1.7	0.09
disagree/disagree on some level/neutral	69	51		41	58	28	43		

Twelve respondents (9%) agreed at least on some level that they are afraid of injuries on artificial turf while 18 respondents (13%) agreed at least on some level to the same statement about natural grass. There were no significant differences between age groups in these statements (Table 2).

Twenty‐eight respondents (28%) considered the quality of the game to be worse on artificial turf. Of these respondents, ten were 11–15‐year‐olds and 18 were 16–20 year‐olds (*Z* = −2.0, *p* = 0.05). Sixty‐seven respondents (49%) agreed at least on some level that artificial turf should not be used if natural grass is available. There was no significant difference between age groups in this statement (Table 2).

**Figure 2:**
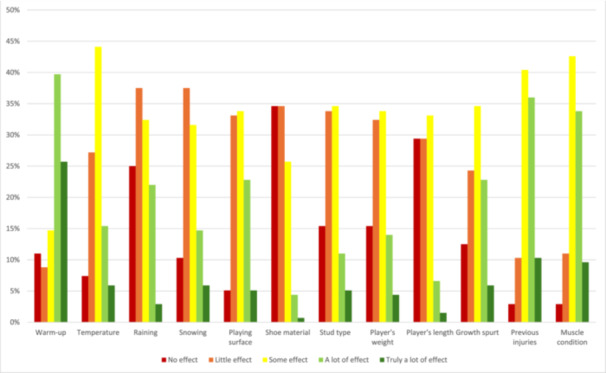
Opinions on the influence of different factors on injury risk (*n* = 136).

When comparing general injury factors, 38 respondents (28%) thought that playing surface has a lot of effect on injury risk, while 52 respondents (38%) thought there is little or no effect. More significant injury factors than playing surface were considered to be warm‐up (a lot of effect in 65% of answers), previous injuries (a lot of effect in 46% of answers), muscle condition (a lot of effect in 43% of answers) and growth spurt (a lot of effect in 29% of answers) (Figure 2, Supplementary Table 2).

**Figure 3:**
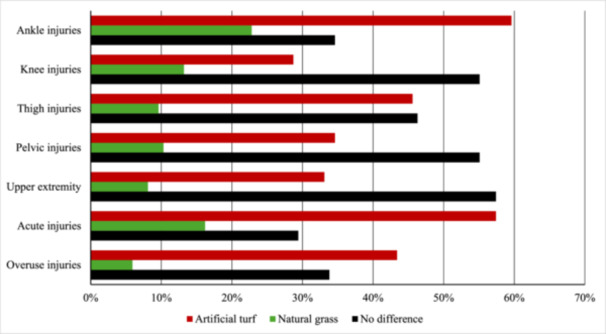
Which surface causes more injuries in the category (*n* = 136).

Artificial turf was seen as a more likely surface to cause injuries in all separate anatomical regions (ankle, knee, thigh, pelvis, upper extremity) and injury types (acute, overuse) compared to natural grass. In anatomical regions, 78 respondents (57%) assessed knee injuries to happen more likely on artificial turf while only 18 respondents (13%) assessed natural grass to cause more knee injuries (*Z* = 4.2, *p* < 0.001). Also, 81 respondents (60%) considered overuse injuries to occur more often on artificial turf, while only eight respondents (6%) considered overuse injuries to occur more often on natural grass (*Z* = 11, *p* < 0.001). In thigh, pelvic and acute injuries, most respondents considered there to be no difference between the surfaces (Figure 3, Supplementary Table 3).

**Figure 4:**
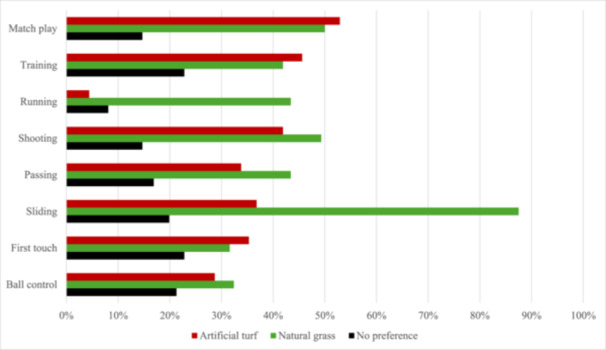
Preferred surface for football actions (*n* = 136).

As a match surface, artificial turf was considered inferior to natural grass (29% vs. 50%, *Z* = −4.0, *p* < 0.001) but as a training surface, there was no significant difference between the surfaces (35% vs. 42%, *Z* = −1.2, *p* = 0.21). While shooting was preferred on natural grass, there was no significant difference in preferences considering running and passing. Artificial turf was considered a significantly worse option for sliding tackles (4% vs. 88%, *Z* = −14, *p* < 0.001). In first touch and ball control, artificial turf was preferred over natural grass (Figure 4, Supplementary Table 4).

**Figure 5:**
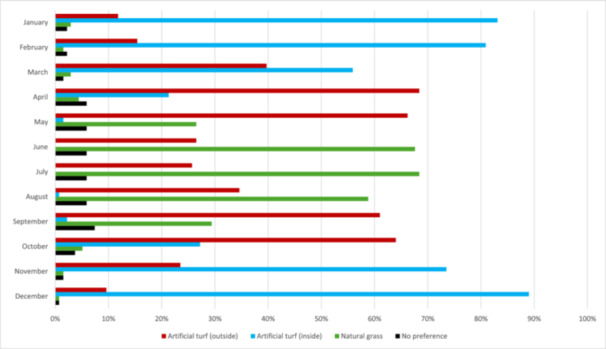
Preferred match surface depending on the month (*n* = 136).

Indoor artificial turf was strongly preferred as a primary playing surface in Finland from November to February (74–89%). Most respondents (56%) also chose inside turf as the primary field in March. Outside artificial turf was the primary choice in April, May, September, and October, while natural grass was preferred only from June to August (Figure 5, Supplementary Table 5).

When pitch condition was considered, most respondents (68%) chose good natural grass as a primary playing surface and most respondents (65%) also chose good artificial turf as the second‐best surface option. As a third option, bad artificial turf was preferred over bad natural grass (51% vs. 32%) (Table 3).

**Table 3:** Preferred match surface in order from best to worst (*n* = 136).

	Best option		Second best option		Third best option		Worst option	
	*n*	*%*	*n*	*%*	*n*	*%*	*n*	*%*
Good natural grass	92	68	26	19	9	7	9	8
Bad natural grass	9	7	12	9	43	32	72	53
Good artificial turf	28	21	88	65	14	10	6	4
Bad artificial turf	7	5	10	7	70	51	49	36

## 4 Conclusion

This survey indicates that players' opinions on the injury risk associated with artificial turf depend on age. In this study population, the younger group of players (11–15‐year‐olds) perceived the injury risk on artificial turf to be higher. Even though the older group of youth players (16–20‐year‐olds) did not consider injuries to increase on artificial turf as much, they did perceive the quality of the game to decrease when playing on artificial surface. In injury subcategories, the risk of knee and overuse injuries was particularly perceived to increase on artificial turf. Natural grass was generally preferred over artificial turf as a primary match surface.

In general, injury risk was considered to increase on artificial surface among the Finnish youth players as nearly half of the respondents perceived injuries to occur more frequently on artificial turf. In previous studies, similar kinds of results have been reported with both amateurs [13] and professionals [15, 16, 20, 21]. However, playing surface was not considered a significant injury factor compared to other possible factors in this study. In a previous questionnaire study, professional players assessed poor pitch quality and artificial turf as the biggest injury factors after poor muscle strength [16] and in other study, even 93‐95% of professional players considered that playing surface has an impact on injury risk [15]. It is notable that the respondents in this study primarily played football on artificial turf throughout the season. Previous studies have found players' perceptions to be dependent on their earlier experience of playing surfaces [13, 14].

In anatomical regions, knee injuries were considered to occur significantly more on artificial turf compared to natural grass. Players also reported overuse injuries to occur more frequently on artificial turf, while most considered there is no difference in acute injury risk. This phenomenon may be emphasized with players who tend to have stress/growth spurt‐related injuries such as Osgood‐Schlatter, which specifically appear in youth athletes. To our knowledge, the impact of artificial turf in overuse injuries has not been properly studied earlier and the topic certainly requires future research. One previous study showed no difference between overuse injuries and surface shifts in professional male football [22].

In this study, first touch and ball control were perceived as better on artificial turf, while shooting was preferred on natural grass. Studies have previously shown that players experience ball interaction to change [18, 23] and tend to be quicker [24] on artificial turf. It is also known that actions in football differ based on the playing surface. For example, fewer sliding tackles [25, 26] and more short passes [25] have been reported to be performed on artificial turf compared to natural grass. Players have also perceived running and other physical efforts to be easier on natural grass [17, 19] and reported artificial turf to be “too fast” [14]. Ball control has also been reported to be worse on artificial turf in a previous study [17]. This aspect is also likely dependent on the previous experience.

Even though Finnish youth players generally preferred natural grass as a match surface, artificial turf (outside or inside) was typically preferred from September to May when weather was taken into account. An average temperature in May and September in Finland is just below 10°C, and rises to roughly 15°C in summer (from June to August) [27]. As a comparison, the divisions of the Football Association of Finland are played outside usually from early April to early November. Considering pitch condition, good natural grass was preferred over good artificial turf but bad artificial turf preferred over bad natural grass. Most natural grass pitches in Finland can realistically be used for football for only a minor part of the year without continuous maintenance, while artificial turf tolerates cold weather conditions well [12, 13].

To our knowledge, this is the first published survey about youth football players' perceptions on artificial turf. Most respondents had played most of their early football career on modern artificial turf and use them regularly all year, which separates our study from previous surveys. We were also able to analyze the impact of age to players' opinions on artificial turf. Despite their young age, the majority of respondents had played football for multiple years and have likely been able to gain experience from various playing surfaces, which can be considered a strength in this study.

The study has limitations. Our population was very homogenous considering respondents' gender and playing history. Therefore, we were unable to analyze differences between some of the subgroups. The lack of female respondents is particularly a limitation in this study, since the evidence of injury risk on artificial turf on certain injury types is more conflicted among them. The respondents were not randomly picked, so it is possible that players with certain opinions about playing surfaces were more eager to respond to this survey. Also, responses were not divided evenly between the participating clubs as VJS was underrepresented considering the size of the club. The quality of playing surfaces was not taken into account, but we know that all the participating clubs had access to at least third‐generation artificial turf. Since the questionnaire was not shared to clubs all over the country, it is possible that the results may not represent the opinions of all youth players in Finland. It is also notable that it is not known how much an average respondent plays football on each surface during a season. In previous studies, players aged 7‐12 years played 65 h of football [5], while Finnish premier division male players played 266 h of football on average during a season [28].

This study shows that there is a conflict between subjective and objective evidence of injury risk on artificial turf in football. It is notable that there are still factors, such as abrasions and heat stress, that are likely to impact on players' perceptions on the surface but require further epidemiological research on modern artificial turf systems [29, 30, 31]. Future research is needed especially with the professionals who have had more negative perceptions on artificial turf in previous studies. In the current 2025 season, 10 of 12 teams in the Finnish male premier division of football had artificial surfaces at their home stadium when the season started. This makes Finland an interesting target for artificial turf research.

## Author Contributions

All authors have read and approved the final version of the manuscript. The corresponding author had full access to all of the data in this study and takes complete responsibility for the integrity of the data and the accuracy of the data analysis.

## Ethics Statement

The authors have nothing to report.

## Consent

All respondents were asked to provide an informed consent for participation.

## Conflicts of Interest

The authors declare no conflicts of interest.

## Transparency Statement

The lead author Ville Immonen affirms that this manuscript is an honest, accurate, and transparent account of the study being reported; that no important aspects of the study have been omitted; and that any discrepancies from the study as planned (and, if relevant, registered) have been explained.

## Supporting information


**Supplementary Table 1.** Current main training surface depending on the season (n=136). **Supplementary Table 2.** Opinions on the influence of different factors on injury risk (n=136). **Supplementary Table 3.** Which surface causes more injuries in the category (n=136). The distribution opinion between playing surfaces was compared using Z‐score with P‐values. **Supplementary Table 4.** Preferred surface for football actions (n=136). The distribution opinion between playing surfaces was compared using Z‐score with P‐values. **Supplementary Table 5.** Preferred match surface depending on the month (n=136).

## Data Availability

The data that support the findings of this study are available from the corresponding author upon reasonable request.
