# Supplementary material for: Finnish Youth Footballers' Perceptions on Artificial Turf: A Survey Research
Source: Health Sci Rep. 2025 Nov 27;8(12):e71594. doi: 10.1002/hsr2.71594 (PMC12660492; doi:10.1002/hsr2.71594)
Supplement: Supplementary file 1 — Supplementary Table 1. Current main training surface depending on the season (n=136). Supplementary Table 2. Opinions on the influence of different factors on injury risk (n=136). Supplementary Table 3. Which surface causes more injuries in the category (n=136). The distribution opinion between playing surfaces was compared using Z‐score with P‐values. Supplementary Table 4. Preferred surface for football actions (n=136). The distribution opinion between playing surfaces was compared using Z‐score with P‐values. Supplementary Table 5. Preferred match surface depending on the month (n=136). [file HSR2-8-e71594-s001.docx]

**Supplementary Table 1.** Current main training surface depending on the season (n=136)

|  | Artificial turf (inside) | | Artificial turf (outside) | | Natural grass | | Other | |
| --- | --- | --- | --- | --- | --- | --- | --- | --- |
|  | *n* | *%* | *n* | *%* | *n* | *%* | *n* | *%* |
| In spring | 14 | 10 | 119 | 88 | 2 | 1 | 1 | 1 |
| In summer | 2 | 1 | 96 | 71 | 38 | 28 | 0 | 0 |
| In autumn | 14 | 10 | 118 | 87 | 4 | 3 | 0 | 0 |
| In winter | 58 | 43 | 78 | 57 | 0 | 0 | 0 | 0 |

**Supplementary Table 2.** Opinions on the influence of different factors on injury risk (n=136)

|  | 1 (no effect) | | 2 (little effect) | | 3 (some effect) | | 4 (a lot of effect) | | 5 (truly a lot of effect) | |
| --- | --- | --- | --- | --- | --- | --- | --- | --- | --- | --- |
|  | *n* | *%* | *n* | *%* | *n* | *%* | *n* | *%* | *n* | *%* |
| Warm-up | 15 | 11 | 12 | 9 | 20 | 15 | 54 | 40 | 35 | 26 |
| Temperature | 10 | 7 | 37 | 27 | 60 | 44 | 21 | 15 | 8 | 6 |
| Raining | 34 | 25 | 51 | 38 | 44 | 32 | 3 | 2 | 4 | 3 |
| Snowing | 14 | 10 | 51 | 38 | 43 | 32 | 20 | 15 | 8 | 6 |
| Playing surface | 7 | 5 | 45 | 33 | 46 | 34 | 31 | 23 | 7 | 5 |
| Shoe material | 47 | 35 | 47 | 35 | 35 | 26 | 6 | 4 | 1 | 1 |
| Button material | 21 | 15 | 46 | 34 | 47 | 35 | 15 | 11 | 7 | 5 |
| Player weight | 21 | 15 | 44 | 32 | 46 | 34 | 19 | 14 | 6 | 4 |
| Player length | 40 | 29 | 40 | 29 | 45 | 33 | 9 | 7 | 2 | 1 |
| Growth spurt | 17 | 13 | 33 | 24 | 47 | 35 | 31 | 23 | 8 | 6 |
| Previous injuries | 4 | 3 | 14 | 10 | 55 | 40 | 49 | 36 | 14 | 10 |
| Muscle condition | 4 | 3 | 15 | 11 | 58 | 43 | 46 | 34 | 13 | 10 |

|  | Artificial turf | | Natural grass | | No difference | |  |  |
| --- | --- | --- | --- | --- | --- | --- | --- | --- |
|  | *n* | *%* | *n* | *%* | *n* | *%* | *Z-score** | *P-value* |
| Ankle injuries | 59 | 43 | 31 | 23 | 46 | 34 | 4.2 | <.001 |
| Knee injuries | 78 | 57 | 18 | 13 | 40 | 29 | 8.7 | <.001 |
| Thigh injuries | 45 | 33 | 13 | 10 | 78 | 57 | 6.0 | <.001 |
| Pelvic injuries | 47 | 35 | 14 | 10 | 75 | 55 | 6.0 | <.001 |
| Upper extremity | 62 | 46 | 11 | 8 | 63 | 46 | 8.4 | <.001 |
| Acute injuries | 39 | 29 | 22 | 16 | 75 | 55 | 3.1 | <.001 |
| Overuse injuries | 81 | 60 | 8 | 6 | 47 | 35 | 11 | <.001 |

**Supplementary Table 3.** Which surface causes more injuries in the category (n=136). The distribution opinion between playing surfaces was compared using Z-score with P-values.
* Z-score compares the answers between the playing surfaces and “no difference” answers were excluded

|  | Artificial turf | | Natural grass | | No preference | |  |  |
| --- | --- | --- | --- | --- | --- | --- | --- | --- |
|  | *n* | *%* | *n* | *%* | *n* | *%* | *Z-score** | *P-value* |
| Matches | 39 | 29 | 68 | 50 | 29 | 21 | -4.0 | <.001 |
| Training | 48 | 35 | 57 | 42 | 31 | 23 | -1.2 | .21 |
| Running | 50 | 37 | 59 | 43 | 27 | 20 | -1.2 | .22 |
| Shooting | 46 | 34 | 67 | 49 | 23 | 17 | -2.8 | .005 |
| Passing | 57 | 42 | 59 | 43 | 20 | 15 | -0.2 | .79 |
| Sliding | 6 | 4 | 119 | 88 | 11 | 8 | -14 | <.001 |
| First touch | 62 | 46 | 43 | 32 | 31 | 23 | 2.6 | .009 |
| Ball control | 72 | 53 | 44 | 32 | 20 | 15 | 3.7 | <.001 |

**Supplementary Table 4.** Preferred surface for football actions (n=136). The distribution opinion between playing surfaces was compared using Z-score with P-values.

* Z-score compares the answers between the playing surfaces and “no preference” answers were excluded

**Supplementary Table 5.** Preferred match surface depending on the month (n=136)

|  | Artificial turf (outside) | | Artificial turf (inside) | | Natural grass | | No preference | |
| --- | --- | --- | --- | --- | --- | --- | --- | --- |
|  | *n* | *%* | *n* | *%* | *n* | *%* | *n* | *%* |
| January | 16 | 12 | 113 | 83 | 4 | 3 | 3 | 2 |
| February | 21 | 15 | 110 | 81 | 2 | 1 | 3 | 2 |
| March | 54 | 40 | 76 | 56 | 4 | 3 | 2 | 1 |
| April | 93 | 68 | 29 | 21 | 6 | 4 | 8 | 6 |
| May | 90 | 66 | 2 | 1 | 36 | 26 | 8 | 6 |
| June | 36 | 26 | 0 | 0 | 92 | 68 | 8 | 6 |
| July | 35 | 26 | 0 | 0 | 93 | 68 | 8 | 6 |
| August | 47 | 35 | 1 | 1 | 80 | 59 | 8 | 6 |
| September | 83 | 61 | 3 | 2 | 40 | 29 | 10 | 7 |
| October | 87 | 64 | 37 | 27 | 7 | 5 | 5 | 4 |
| November | 32 | 24 | 100 | 74 | 2 | 1 | 2 | 1 |
| December | 13 | 10 | 121 | 89 | 1 | 1 | 1 | 1 |
